# Binding Mode Characterization of Osteopontin on Hydroxyapatite by Solution NMR Spectroscopy

**DOI:** 10.1002/cbic.202100139

**Published:** 2021-05-10

**Authors:** Julian Holzinger, Harald Kotisch, Klaus W. Richter, Robert Konrat

**Affiliations:** ^1^ Department of Structural and Computational Biology University of Vienna, Max Perutz Labs Vienna BioCenter Campus 5 1030 Vienna Austria; ^2^ Vienna Biocenter Core Facilities GmbH Dr. Bohr Gasse 3 1030 Vienna Austria; ^3^ Department of Inorganic Chemistry, Functional Materials University of Vienna Währinger Str. 42 1090 Vienna Austria

**Keywords:** biomineralization, hydroxyapatite, intrinsically disordered protein, NMR, osteopontin, phosphorylation

## Abstract

Extracellular matrix glycoproteins play a major role in bone mineralization and modulation of osteogenesis. Among these, the intrinsically disordered protein osteopontin (OPN) is associated with the inhibition of formation, growth and proliferation of the bone mineral hydroxyapatite (HAP). Furthermore, post‐translational modifications like phosphorylation can alter conformations and interaction properties of intrinsically disordered proteins (IDPs). Therefore, the actual interaction of OPN with a HAP surface on an atomic level and how this interaction is affected by phosphorylation is of great interest. Here, we study the interaction of full‐length OPN on the surface of suspended HAP nanoparticles by solution NMR spectroscopy. We report the binding modes of this IDP and provide evidence for the influence of hyperphosphorylation on the binding character and an explanation for the differing roles in biomineralization. Our study moreover presents an easy and suitable option to measure interaction of nanoparticles in a stable suspension with full‐length proteins.

## Graphical abstract

**The intrinsically disordered protein** osteopontin is associated with modulating the formation, growth and proliferation of the bone mineral hydroxyapatite. Employing different solution NMR spectroscopy methods, we characterize the binding modes to hydroxyapatite and provide evidence for the influence of phosphorylation of the protein on the binding character and the differing roles in biomineralization.

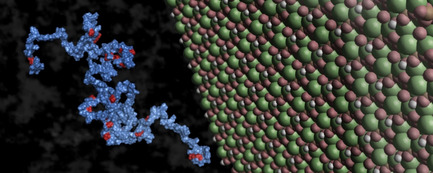

## Introduction

Human osteopontin (OPN) is an intrinsically disordered protein (IDP), also known as SPP1 (secreted phosphoprotein 1). It is a secreted and chemokine‐like extracellular glycoprotein and belongs to the SIBLING (small integrin‐binding ligand N‐linked glycoprotein) family.^[1]^ It has an average molecular weight of 44 kDa, is highly negatively charged due to the large number of glutamic and aspartic acid residues, and is regulated by post translational modifications like glycosylationand phosphorylation.^[2]^ OPN exhibits multiple physiological functions, as well as versatile pathological effects. It acts as a chemokine (binding to integrin receptors and CD44; modulator of cell adhesion and migration) and binds and activates metalloproteases.[^3^, ^4^] Furthermore, it is reported to be implicated in biological processes like apoptosis, angiogenesis, cell proliferation, wound healing and tissue remodeling.[^3^, ^4^, ^5^, ^6^, ^7^] Due to its ability to influence cell migration, it is reported to function as an auto‐ and paracrine mediator of tumor growth, progression and metastasis.[^8^, ^9^, ^10^, ^11^, ^12^] However, most of all, OPN is associated with bone mineralization and the modulation of osteogenesis. It was reported to be important in the differentiation and recruitment of osteogenic cells, especially in the interaction with osteoclasts,[^13^, ^14^, ^15^, ^16^, ^17^, ^18^, ^19^, ^20^] and as an inhibitor of hydroxyapatite (HAP; Ca_10_(PO_4_)_6_(OH)_2_; principle component of bone: approx. 65 wt%) formation, growth and proliferation,[^21^, ^22^, ^23^, ^24^, ^25^] whereas an OPN deficiency can cause dystrophic calcifications in mice and humans and a decline in fracture/bone toughness.[^19^, ^22^, ^26^, ^27^] In recent studies OPN was found to stabilize a transient pseudo‐coacervate phase of calcium phosphate in a saturated solution, enabling the entrance of these droplets into the collagen fibers and thus promoting intrafibrillar mineralization, and finally moderating extrafibrillar mineral coating.[^23^, ^28^, ^29^, ^30^, ^31^] Due to its high amount of negatively charged residues and its ability to get hyperphosphorylated, an adsorption of probably electrostatic nature on a mineral surface is very likely, which leads to an inhibitory effect on mineral growth.[^19^, ^32^] Moreover, the intrinsically disordered nature of OPN is also suggested to facilitate the adsorption on a mineral surface due to the possibility of multiple binding site formations.^[32]^ Both a conformational energy change upon binding to a mineral surface^[33]^ and the flexible nature of the structure when bound to a HAP surface were shown by computational methods.^[34]^ However, a detailed picture of the interaction of OPN with the surface of HAP has yet not been determined.

Phosphorylation regulates the binding interaction of OPN with HAP and therefore also mediates HAP formation and growth.[^35^, ^36^] It was shown that the phosphorylated isoform is a stronger inhibitor of HAP formation than the non‐phosphorylated form of HAP.[^32^, ^33^, ^36^, ^37^] Besides that, hyperphosphorylation also has an impact on structural dynamics and molecular recognition of OPN (e. g. the binding to extracellular matrix components and integrin receptors).[^38^, ^39^] Fam20 C (family with sequence similarity 20, member C) kinase has been identified to phosphorylate serine and threonine residues of secreted proteins in the Golgi apparatus, among others OPN.[^40^, ^41^] Mutations of this kinase affect its activity and cause a bone dysplasia characterized by ectopic calcifications and osteosclerosis, called Raine syndrome, which in many cases is fatal in the neonatal period.[^42^, ^43^] Fam20 C recognizes and phosphorylates S‐x‐E/pS motifs, whereby phosphorylation of other motifs has been reported as well.[^38^, ^39^, ^44^] OPN contains 22 of these motifs, and thus, in case of a full phosphorylation, it is hyperphosphorylated. Recently we identified the phosphorylation of 28 sites in OPN by Fam20 C via a combined approach of NMR spectroscopy and mass spectrometry.^[39]^

## Experimental Section

**Figure 1:**
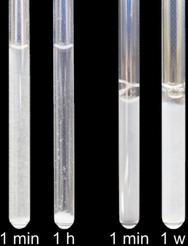
NMR tubes with HAP in buffer (left) and MeCe(1.6 wt%) hydrogel (right) after preparation, 1 h and 1 week, respectively.

We investigated the interaction of OPN and OPNp (hyperphosphorylated OPN) with Ca^2+^ ions in solution, and further, the interactions of OPN(p) with synthesized HAP nanoparticles (for synthesis and characterization see SI S4, Figures S1 and S2) by solution NMR spectroscopy. NMR spectroscopy has proven to be a powerful tool in structural biology, especially for describing structures and dynamics of IDPs. The characterization of the protein‐nanoparticle interaction depends on the exploitation of multidimensional NMR experiments for the discrimination of signals in an isotopically labelled protein sample. These kinds of experiments require a major amount of time which is why sedimentation of the nanoparticles inside of a NMR tube and thus a proper determination of the interaction becomes an issue. If the nanoparticles are not small enough to create a stable suspension in the measurement buffer,[^45^, ^46^] aqueous gels can be employed to prevent nanoparticle sedimentation and to provide stable homogeneous suspensions. For this purpose, Egner et al.^[47]^ and Xue et al.^[48]^ used e. g. a 1 wt% agarose gel for describing the interaction of cholic acid and phenol with CeO_2_ and different amino acids with TiO_2_ nanoparticles, respectively. In this study, methylcellulose (MeCe) hydrogel was chosen as a chemically inert matrix. This viscous hydrogel exhibits large porosity, which allows water‐soluble compounds to tumble freely in solution while at the same time it prevents sedimentation, and it has proven not to interact with the proteins investigated, as shown in a study of OPN binding to integrin receptors in living cells.^[49]^ Herein, we show that 0.6 mg of HAP nanoparticles can be suspended in 600 μL of a 1.6 wt% MeCe gel (=0.1 wt% HAP) for obtaining a homogeneous suspension without sedimentation over several days – in comparison to the same amount of HAP in the measurement buffer only where sedimentation sets in right after the initial suspension (Figure 1).

## Results and Discussion

### Electrostatic binding mode of OPN(p) to Ca^2+^ cations

**Figure 2:**
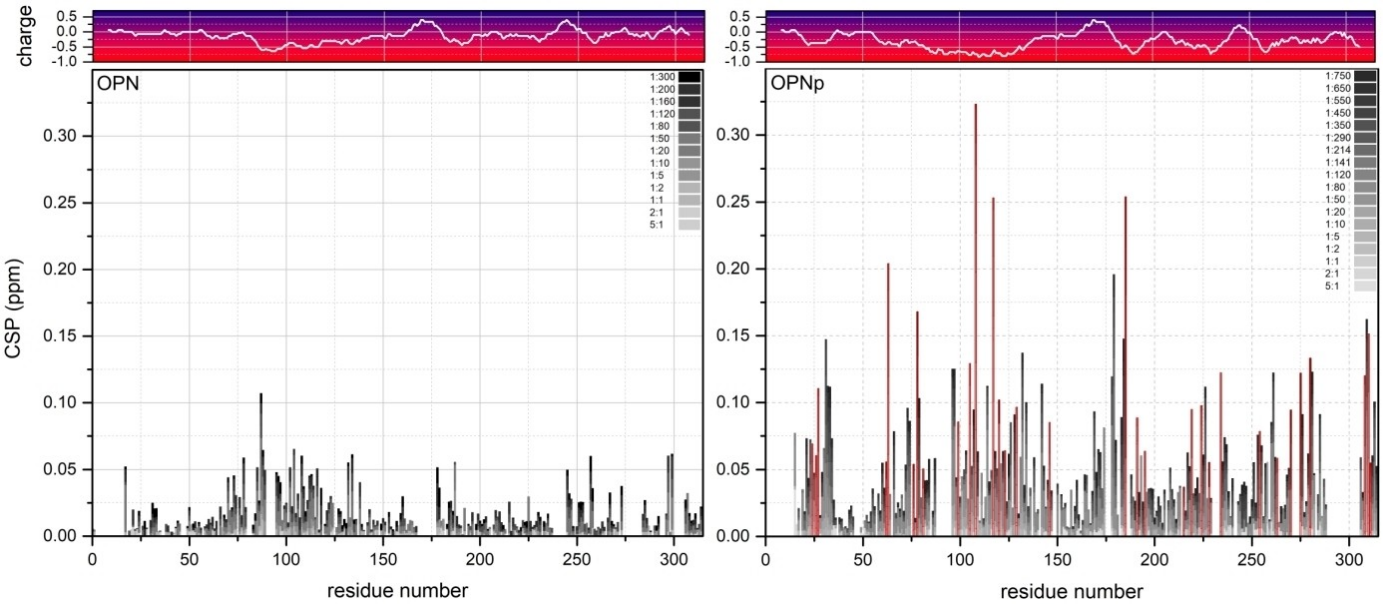
Titration series and chemical shift perturbations (CSP) of OPN (left) and OPNp (right) with CaCl_2_, including the corresponding charge plots on top. The grayscale labels the increasing molar ratio of OPN : Ca^2+^, from 5 : 1 to 1 : 300 (non‐phosphorylated) and 5 : 1 to 1 : 750 (phosphorylated). Phosphorylated residues are marked in red.

Binding of Ca^2+^ to both the unphosphorylated and phosphorylated form of OPN was investigated by titration of CaCl_2_ and following the corresponding chemical shift perturbations from ^1^H‐^15^N HSQC NMR spectra. Binding affinities were determined by fitting the chemical shift perturbations (CSP) of the most pronounced perturbations to an analytical function (Figures S3 and S4), as suggested by Williamson.^[50]^ OPN shows Ca^2+^‐binding mainly in the aspartate‐rich regime (poly‐D regime; 85–116), accompanied by less pronounced perturbations in the other negatively charged regions 70–79, 130–136, 178–188 and 245–260 (Figure 2, left), revealing binding affinities in the lower millimolar range (4–16(3) mM). Due to the phosphorylated sites, OPNp in total carries a higher negative charge than the unphosphorylated homolog and the negatively charged regions are distributed broader. Chemical shift perturbations of OPNp binding to Ca^2+^ are mainly observed in the regions 96–137 (including seven phosphoserines and the poly‐D regime), 174–185 and 306–311 (Figure 2, right). Additionally, perturbations are also observed in other regions, such as 21–35, 72–81, 217–238 and 268–281. An analysis of the most pronounced perturbations again resulted in affinities in the lower millimolar range (2–20(3) mM).

According to the size of perturbations, the binding of OPN to Ca^2+^ cations seems stronger upon phosphorylation, however the binding affinities are in the same range. Moreover, a higher amount of Ca^2+^ cations was needed to reach saturation. These findings are in agreement with binding studies of rat and bovine OPN, where binding affinities of 1–3 mM and a high Ca^2+^ binding capacity were observed[^51^, ^52^] (in contrast to the mid‐nanomolar range affinity (30–50 nM) reported by Kläning et al.^[53]^ – however their human OPN sample had been EDTA‐dialysis‐treated and thus was completely Ca^2+^‐free). Considering the high binding capacity and following the idea of Kläning et al.^[53]^ that it takes several binding sites to ‘chelate’ the Ca^2+^ cation, the reported stoichiometry of 8–12 Ca^2+^ per OPNp[^53^, ^54^] matches our observation. Both the low affinity and the high binding capacity are consistent with a predominantly electrostatic binding mode of OPN(p) to Ca^2+^ cations.

### Different binding modes of OPN(p) on HAP surface

Binding of OPN(p) to the surface of HAP nanoparticles and the differences compared to the apo‐form were investigated employing ^1^H‐^15^N HSQC NMR (chemical shift perturbations), ^15^N−R_2_ relaxation NMR (structural dynamics), STD NMR (saturation transfer difference; ligand‐receptor interaction) and ^1^H^N^ PRE NMR (paramagnetic relaxation enhancement; long‐range structural interaction) experiments. Additionally, adsorption isotherms for OPN(p) adsorbing onto HAP nanoparticles were determined (SI S8 and Figure S5). Dissociation constants $K_{D}$ for OPN and OPNp of 28(17) μM and 14(4) μM, and monolayer sorption saturation capacities Χ_m_ of 0.011(3) μmol/mg and 0.006(1) μmol/mg, respectively, were obtained by fitting the data to a Langmuir isotherm.

**Figure 3:**
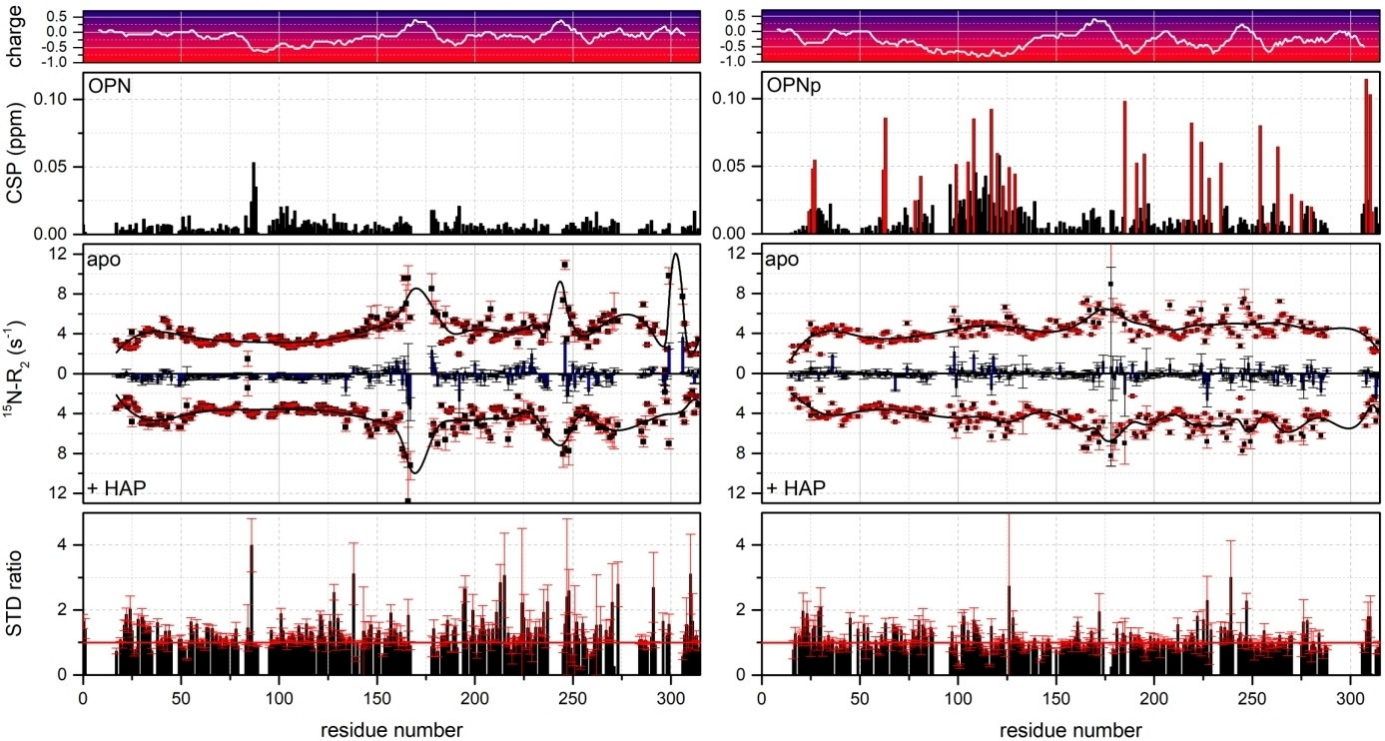
Comparison of OPN (left) and OPNp interacting with HAP nanoparticles. Chemical shift perturbations (CSP) with HAP (0.1 wt%) in MeCe (1.6 %), phosphorylated residues in OPNp are marked in red (top); ^15^N−R_2_ relaxation parameters of the apo form and with HAP, including the ^15^N−R_2_ differences (blue bars) (middle). STD ratios (+HAP/apo ; the red line indicates a STD ratio of 1, i. e., no STD effect) (bottom). The corresponding charge plot of the protein sequences is included at the very top.

Similar to the binding to Ca^2+^ cations, both OPN and OPNp show CSPs mainly in the negatively charged regions upon the interaction with HAP surfaces (Figure 3, top). Again, OPN binds to HAP predominantly in the poly‐D regime (residues 85 ff.). OPNp interactions with HAP are mainly detected in the large negatively charged patch between residues 96–137, including the poly‐D regime and several phosphoserines, the N‐ (21–35) and the C‐terminus (306–311) regions and three regions in the second half of the protein (∼190, ∼220 and ∼260). Most of the phosphoserines reveal pronounced perturbations and therefore the interactions sites of OPNp on the mineral surface are distributed over the whole protein length. In both forms, the integrin‐binding domain (^159^RGD^161^) is unaffected upon binding to HAP. The ^15^N relaxation rates (Figure 3, middle) of the apo forms of OPN(p) were discussed recently,^[38]^ showing an overall decrease in ^15^N transverse relaxation rates and thus an increase in backbone dynamics in the nanosecond timescale for the region 200–314 upon phosphorylation, and slightly less dynamics in the region 96–137 (large negatively charged area in OPNp). However, no significant changes in ^15^N−R_2_ values neither for OPN nor OPNp are observed upon the interaction with HAP nanoparticles, suggesting that the protein is not greatly immobilized on the HAP surface. Considering the absolute values of the CSPs and especially the similarity of the ^15^N−R_2_ values for both OPN and OPNp upon interaction with the mineral surfaces, the binding of OPN(p) to HAP in liquid seems to be of a low‐affinity nature, as tight binding events to a nanoparticle would lead to a considerable increase in transverse relaxation rates. Like for the binding to Ca^2+^ cations, this is consistent with a predominantly electrostatic binding mode of OPN(p) to HAP nanoparticle surfaces.

STD NMR methods exploit the intermolecular magnetization transfer between target and ligand spins in transiently forming complexes by saturating the target resonance selectively and transferring this saturation to the contacting ligand spins which results in a decreased line intensity of the latter. We chose an on‐resonance saturation pulse of 1 s and 1.8 kHz at −5 ppm which should cover the resonances of the surface hydroxyl groups of HAP in solution NMR spectroscopy. However no major STD effects were detectable in the presence of HAP, neither for OPN nor OPNp (Figure 3, bottom) – in comparison to the STD effects of OPN to HEK293T cells in MeCe.^[49]^ The absence of a major STD effect may not be too surprising considering the low amount of protons on the surface of HAP and the weak interaction of OPN(p) to it. Nevertheless, small STD effects are detected in the second half of OPN upon binding to HAP (Figure 3, bottom left). These effects might originate from increased intramolecular interactions of the residues located in the main compact state of OPN upon binding. In comparison, no considerable STD effects are observable in the case of OPNp (Figure 3, bottom right) reflecting the conserved expanded state of OPNp even upon binding to HAP surfaces.

**Figure 4:**
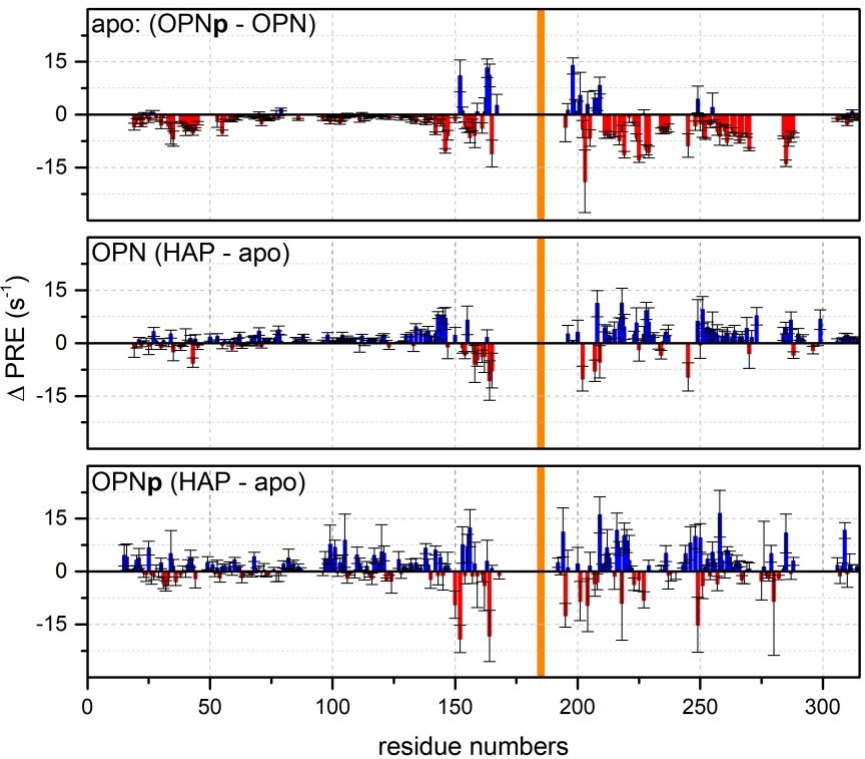
Long‐range interactions measured by PRE experiments of the OPN(p) cysteine mutant T185 C, determined from ^1^H^N^−T_2_ NMR experiments. PRE rates difference (Δ PRE=Γ_2_(OPNp)‐ Γ_2_(OPN)) of OPN and OPNp (top) as reported before^39^. PRE rates difference of OPN with HAP and in the apo form (middle). PRE rates difference of OPNp with HAP and in the apo form (bottom).

In order to further investigate these long‐range interactions of the two protein forms upon binding to HAP surfaces, PRE NMR experiments of the cysteine mutant T185 C of both forms were performed in the apo and the HAP‐bound state (Figures 4, S6 and S7). As we have reported recently,^[39]^ the apo form of the OPNp shows a remarkable reduction of long‐range interactions within the main compact state and towards the N‐terminal region in comparison to the unphosphorylated OPN (Figure 4, top), suggesting that the phosphorylated form has a significantly elongated structure. In the presence of HAP nanoparticles, OPN shows a clearly enhanced PRE effect within the central core region, however not towards the N‐terminal region (Figure 4, middle). A PRE rates correlation map (Figure S8, left) indicates a rather correlated enhancement of the PRE effect of the main compact state in the apo and the HAP‐bound sample. Considering the observed binding of OPN to HAP at the poly‐D regime (residues 85 ff.), the increased PRE effect upon binding and the correlation suggest that the second half of the OPN remains in the compact state and is still tumbling freely but with a longer τ_ex_ and hence not interacting with the HAP surface. Likewise, the phosphorylated OPNp shows mainly enhanced PRE effects upon the interaction with HAP (Figure 4, bottom). However, in this case the enhanced PRE effects appear at the main binding domain (residues 96–137) and two binding regions in the second half of the protein (∼220 and ∼260), annotating that the MTSL label is located in the vicinity of one binding domain around the residue 190. In contrast to the unphosphorylated OPN, the PRE rates correlation map (Figure S8, right) of the phosphorylated protein indicates a rather uncorrelated enhancement of the PRE effect from the apo to the HAP‐bound state, meaning that the bound state is decoupled from any structural substates. These findings suggest that OPNp remains in its elongated state upon binding to HAP, as indicated by the STD NMR results (*vide supra*), however somewhat more rigid at the binding domains around the residues 96–137, ∼190, ∼220 and ∼260, which leads to the observed PRE effects. The binding regions at the N‐ (21–35) and C‐terminus (306–311) regions do not reveal a major PRE effect upon binding to HAP, suggesting that the protein termini do not undergo this slight rigidification upon binding like the other binding domains.

To summarize, the data suggests that OPNp – more elongated structure, broadly distributed phosphoserines – binds to HAP surfaces over the full length remaining elongated and covering the mineral surface. In contrast, the non‐phosphorylated OPN binds more locally and weaker. These findings are supported by the differences in $K_{D}$ (28 *vs* 14 μM) and Χ_m_ (0.011 *vs* 0.006 μmol/mg) for OPN and OPNp upon binding to HAP (stronger binding of OPNp, higher coverage of OPNp and thus lower saturation capacity; SI S8 and Figure S5). Furthermore, the central part of the protein containing the RGD‐motif (^159^RGD^161^) neither in OPN nor in OPNp shows any chemical shift perturbations or PRE effects upon binding to HAP, hence it is still available to interact with integrin receptors.

### OPN(p) on HAP surface – comparison to other HAP‐binding proteins and function

In the past, the interaction of two other calcium phosphate proteins, statherin and amelogenin, on the surface of HAP has been investigated by solid‐state NMR spectroscopy, revealing two quite different binding modes. Statherin was found to form a tight helix in the C‐terminal region at the protein‐surface interface induced upon binding to HAP. It binds very locally, including two phosphoserines adjacent to a D and two E residues, and revealing a latter‐matching mechanism.[^55^, ^56^, ^57^, ^58^, ^59^] On the contrary, amelogenin and its splice variant LRAP (Leucine‐rich amelogenin peptide) exhibit two rather unstructured HAP‐binding regions at the N‐ and C‐terminal regions, largely extended in order to optimize electrostatic interactions with the mineral surface and with a significant amount of motion. Upon phosphorylation, the binding domain is even more extended and the backbone closer to the mineral surface.[^60^, ^61^, ^62^] Especially the binding mode of amelogenin on HAP shows close resemblance to the here studied binding mode of OPNp on HAP.

These described binding modes of OPN(p) are in accordance with their reported differing roles in biomineralization. OPNp has been shown to be a natural inhibitor of calcification and mineral formation, growth and proliferation,[^19^, ^21^, ^22^, ^32^, ^35^] and to prevent crystal formation in soft tissues and biological fluids.[^25^, ^28^, ^32^] Whereas the non‐phosphorylated form always has shown less or no effect (e. g. recently, a weak binding of non‐phosphorylated quail OPN on HAP crystal surfaces has only mildly affected the crystallite properties^[63]^). This difference in function becomes clear by considering the elongated structure (in the apo and HAP‐bound state) of OPNp binding over the full length hence covering a larger surface area. Additionally, at the same time it has a higher affinity to HAP than the non‐phosphorylated OPN. The same holds for the stabilization of crystallization intermediates in saturated solutions, also called transient pseudo‐coacervate phases of calcium phosphates,[^28^, ^29^, ^30^] which may serve as a process‐directing agent for intrafibrilliar mineralization in collagen:[^23^, ^31^] OPNp is capable of stabilizing these phases due to the elongated and more flexible structure, its higher binding capacity to Ca^2+^ and higher affinity to HAP.

Regarding the role of the RGD‐motif interacting with bone cells, on the one hand a decreased binding affinity of OPNp to integrin receptors in comparison to the non‐phosphorylated form has been reported.[^38^, ^44^] On the other hand however, OPNp has been reported to play a role in initial osteoclast recognition and attachment to bone by ‘anchoring’ osteoclasts in the bone surface via an integrin receptor, also revealing an increased binding to osteoclasts *in vitro*.[^13^, ^14^, ^15^, ^19^, ^20^, ^23^] From our data we can only speculate that the tighter binding of OPNp to the HAP surface while providing an unaltered integrin‐binding RGD‐motif adds an explanation to its participation in bone remodeling.

## Conclusion

We investigated the binding of OPN to HAP nanoparticle surfaces and the impact of phosphorylation. The application of MeCe has shown to be an easy and suitable option to keep the nanoparticles in a stable suspension over several days, to maintain a solution‐like environment for the protein and not to interact with the full‐length protein investigated. NMR studies on OPN(p) upon binding to HAP revealed different binding modes. The unphosphorylated OPN binds weakly, predominantly in the poly‐D regime (residues 85 ff.), to HAP surfaces, while its main compact state is not interacting with the HAP surface and remains unaltered. By contrast, the OPNp, which has a more elongated structure due to a reduction of long‐range correlations by the hyperphosphorylation, binds to HAP surfaces over the full length as phosphorylated residues are distributed broadly over the structure, especially in the domains at residues 96–137, ∼190, ∼220 and ∼260. Furthermore, the data suggest that the structure of OPNp remains elongated upon binding, covering the HAP surface. For both OPN and OPNp the binding to HAP surfaces is of an electronegative nature and no conformational changes can be observed upon binding. Furthermore, the central part containing the RGD‐motif does not participate in the binding event and thus is still available to interact with integrin receptors. However, the different binding modes may explain the distinct biological functions of OPN and OPNp during osteogenesis and biomineralization: the rather “covering” binding mode of OPNp may explain its function as a mineralization inhibitor through physically blocking the mineral surface from further growth[^19^, ^21^, ^23^, ^32^] and its property to stabilize transient pseudo‐coacervate phases of calcium phosphate.[^23^, ^25^, ^28^, ^29^]

## Accession Codes

Human OPN: P10451 (Uniprot); OPNp: P50447 (BMRB); Human Fam20 C: Q8IXL6 (Uniprot).

## Conflict of interest

The authors declare no conflict of interest.

## Supporting information

As a service to our authors and readers, this journal provides supporting information supplied by the authors. Such materials are peer reviewed and may be re‐organized for online delivery, but are not copy‐edited or typeset. Technical support issues arising from supporting information (other than missing files) should be addressed to the authors.

Supplementary
