# Supplementary material for: Binding Mode Characterization of Osteopontin on Hydroxyapatite by Solution NMR Spectroscopy
Source: Chembiochem. 2021 May 10;22(13):2300–5. doi: 10.1002/cbic.202100139 (PMC8359842; doi:10.1002/cbic.202100139)
Supplement: Supplementary file 1 — Supplementary [file CBIC-22-2300-s001.pdf]

ChemBioChem

Supporting Information

Binding Mode Characterization of Osteopontin on Hydroxyapatite by Solution NMR Spectroscopy

Julian Holzinger,* Harald Kotisch, Klaus W. Richter, and Robert Konrat

Supporting Information

S1 Expression of human OPN

Uniformly ^{15}N-labelled OPN (residue 17 to 314 of the full-length protein; BMRB ID: P10451) was expressed and purified in its non-phosphorylated form as reported previously[1,2]. As a final step, the buffer was exchanged to the desired measurement buffer (BisTris 50 mM, NaCl 50 mM, NaN_3 1 mM, pH=6.5).

```
                      IPVK QADSGSSEEK QLYNKYPDAV ATWLNPDPSQ
KQNLLAPQNA VSSEETNDFK QETLPSKSNE SHDHMDDMDD EDDDDHVDSQ

DSIDSNDSDD VDDTDDSHQS DESHHSDESD ELVTDFPTDL PATEVFTPVV
PTVDTYDGRG DSVVYGLRSK SKKFRRPDIQ YPDATDEDIT SHMESEELNG

AYKAIPVAQD LNAPSDWDSR GKDSYETSQL DDQSAETHSH KQSRLYKRKA
NDESNEHSDV IDSQELSKVS REFHSHEFHS HEDMLVVDPK SKEEDKHLKF

RISHELDSAS SEVN
```

S2 Phosphorylation of OPN

^{15}N OPN was phosphorylated *in vitro* employing the kinase Fam20C. Fam20C was expressed as described previously[1,3]. The phosphorylation was performed as described previously[1] (BMRB ID: P50447).

S3 MTSL labelling

The ^{15}N OPN(p) cysteine mutant T185C were incubated with an excess of DTT (10 mM; 15min, RT). A PD-10 Desalting-Column was equilibrated with 100 mM sodium phosphate buffer (1 mM EDTA, pH = 8.0), the protein loaded (V_{tot} = 2.5 mL) and eluted with 3.4 mL of buffer. Free thiol concentration was checked using a 300 mM DTNB (5,50-dithiobis-(2-nitrobenzoic acid)) solution at 412 nm. A threefold excess of *S*-(1-oxyl-2,2,5,5-tetramethyl-2,5-dihydro-1H-pyrrol-3-yl) methyl methane-sulfonothioate (MTSL) was added and the sample was incubated under agitation (3 h, 37 ºC). Afterwards, the free thiol concentration was checked again. Protein purity was verified via SDS-PAGE electrophoresis. MTSL is a nitroxide spin radical and thus the MTSL-labelled protein serves as the paramagnetic form. The nitroxide group is reduced with ascorbic acid in order to obtain the diamagnetic form.

S4 Synthesis and Characterization of Hydroxyapatite Nanoparticles

S4.1 Synthesis. Hydroxyapatite (HAP) nanoparticles were synthesized by precipitation titration, similar to Matlahov et al.[4]. 16 mL of $(NH_4)_2PO_4$ (0.075 M) were titrated into 56 mL solution consisting of $Ca(NO_3)_2$ (0.04 M) and NH_3 (0.2 M / 0.8%) at 37°C and pH=9, using a peristaltic pump (0.05 mL/min), and under continuous stirring (850 rpm). After the complete add of the phosphate solution, the mixture was stirred overnight at 60°C. The solid precipitate was filtered (0.45 µm), washed with ethanol and MilliQ water and dried at 37°C for 48h. The obtained HAP was ground finely in a porcelain mortar and stored as it is. For comparison and to confirm the right sample composition in powder XRD measurements, one HAP sample was calcined at 900°C for 24h.

S4.2 pXRD. Powder XRD patterns were obtained with a Bruker D8 Advance diffractometer (Cu Kα) in pseudo-focussing Bragg-Brentano configuration using variable slits and a silicon strip detector (Lynxeye). Data were analyzed with the TOPAS software[5] and a structural model of the hexagonal HAP (space group $P6_3/m$). Powder XRD diffractograms of the

synthesized HAP nanoparticles, the calcined HAP and the corresponding Rietveld refinements are shown in **Figure S1**. Background corrections, an amorphous foreign phase peak at 62.48° and four reflections of impurities (15.02°, 30.29°, 37.36° and 46.12°) are included in the refinements, resulting in R-values of R_{wp} = 4.11 (calcined HAP) and R_{wp} = 6.35 (HAP nanoparticles). The calcined sample exhibits sharp reflections (refined average crystalline domain size: 72 nm) at the positions of hexagonal HAP, revealing that the annealing at high temperature produced a highly crystalline HAP. The pattern of the HAP nanoparticles shows broader reflections that can be refined with the same hexagonal structure. The larger peak widths are caused by the average crystallite size of 12 nm (determined with the Scherrer equation). The peaks of the unknown impurity phase correspond either to a simple cubic cell (a = 5.898 Å; 001, 002, 211 and 003 reflexes) or a body centered cubic cell (a = 8.340 Å; 011, 022, 222 and 411 reflexes) according to an indexing attempt.

S4.3 Transmission Electron Microscopy. HAP nanoparticles were suspended and sonicated in ethanol, and deposited on 200 mesh copper R 1.2/1.3 Quantifoil holey carbon grids (Großlöbichau, Germany) for TEM with an additional 2 nm continuous carbon film. After the solvent had evaporated grids were imaged using a Thermo Scientific Glacios transmission electron microscope operated at 200 kV and micrographs were digitally recorded using a Falcon3 direct electron detector in linear mode. Images of the HAP nanoparticles are shown in **Figure S2**. The nanoparticles appear in clusters of seemingly detachable crystallites of platelet-like morphology with an approximate size of 10-20 nm by 5 nm, which is in accordance with the determined crystallite size from the pXRD pattern refinement. Crystal planes can be observed in some crystallites in the micrograph upon the highest magnification (**Figure S2**, bottom).

S5 Preparation of MeCe Hydrogel

The commercially available Methocel® A4M (Sigma-Aldrich) was dispersed in the pre-heated (80°C) measurement buffer (BisTris 50mM, NaCl 50mM, NaN_3 1uM, pH=6.5). The mixture was stirred for 15min until the MeCe was thoroughly wetted and in suspension. Afterwards, more buffer was added to the mixture to reach the desired concentration of 2 wt% and it was incubated at 4°C overnight under agitation.

S6 Preparation of NMR Sample

S6.1 Samples in MeCe. 480µL of MeCe (2wt%) were mixed with 60µL D_2O and 60µL of the respective protein solution (final concentration: 0.08 – 0.1 mM for wt; 0.03 – 0.05 mM for T185C mutant), resulting in a MeCe concentration of 1.6 wt%.

S6.2 Samples w/ HAP. 0.6 mg HAP were suspended in 480µL of the MeCe hydrogel/measurement buffer + 60µL D_2O applying 3x1' of sonication (70 W; 30% amplitude; 1s/1s on/off duty cycle), resulting in a HAP concentration of 0.1 wt%. Afterwards, the suspension was extensively mixed with 60µL of the respective protein solution.

S7 NMR Experiments

NMR spectra have been acquired at 293K on a Bruker Avance III HD+ 800 spectrometer (18.8 T), using a Bruker RT 5mm TXI probe. ^{15}N-T_2 relaxation data was determined using CPMG delays of 0.017, 0.034, 0.136, 0.271 and 0.543 s. For the STD ^{1}H-^{15}N HSQC experiments, an irradiation pulse (1s; 1.8 kHz) was employed at -5 ppm and -50 ppm for the on- and off-resonance experiment, respectively. For the PRE measurements, $^{1}H^{N}$-R_2 rates for hOPN(p) T185C were determined employing a pseudo 3-D $^{1}H^{N}$-T_2 HSQC NMR. Six relaxation delays (0.001, 0.005, 0.01, 0.02, 0.05 and 0.1 s) were used in order to obtain the relaxation rates from exponential decay function fits.

NMR data was processed with nmrPipe[6] and analysed with Sparky[7]. Chemical shift perturbations (CSP) were read out from the respective ^{1}H-^{15}N HSQC spectra and calculated according to:

$$\mathrm{CSP} = \Delta\delta = \sqrt{(\Delta\delta_{\mathrm{H}})^2 + (\Delta\delta_{\mathrm{N}}/5)^2}$$

Binding affinities of Ca^{2+} to OPN(p) from titration NMR experiments (**Figure S3** and **S4**) were analyzed and fitted with a low-binding event equation[8] from which the affinities K_{D} were determined (c(OPN) = 0.1 mM; c(OPNp) = 0.05 mM):

$$\mathrm{CSP} = \Delta\delta_{\max}\{([P]_{\mathrm{tot}} + [L]_{\mathrm{tot}} + K_{\mathrm{D}}) - [([P]_{\mathrm{tot}} + [L]_{\mathrm{tot}} + K_{\mathrm{D}})^2 - 4[P]_{\mathrm{tot}}[L]_{\mathrm{tot}}]^{1/2}\} / 2[P]_{\mathrm{tot}}$$

Figures S3 and **S4** show the fit analysis performed for selected sets of residues from the regions with the largest chemical shift perturbations.

The STD effect (ΔSTD) was quantified by a normalization of the spectral intensities by running the same experiment with and without the saturation pulse (STD_x and HSQC_x) and under the compared conditions (*a* and *b*; e.g. +HAP and apo). It was calculated as follows:

$$\Delta\mathrm{STD} = (\mathrm{STD}_a/\mathrm{HSQC}_a)\ /\ (\mathrm{STD}_b/\mathrm{HSQC}_b)$$

The PRE effect ($\Delta(^1\mathrm{H}^{\mathrm{N}})\Gamma_2$) is quantified according to:

$$\Delta(^1\mathrm{H}^{\mathrm{N}})\Gamma_2 = (^1\mathrm{H}^{\mathrm{N}})R_{2,\mathrm{para}} - (^1\mathrm{H}^{\mathrm{N}})R_{2,\mathrm{dia}}$$

S8 Adsorption Isotherms (OPN(p) + HAP)

Known initial concentrations of OPN(p) (c_0 = 0.004 – 0.08 mM; V_0 = 0.4 mL) were equilibrated with 0.6 mg of HAP nanoparticles for 4h under agitation. Nanoparticles were separated from the supernatant by centrifugation (13k rpm, 20 min). The equilibrium protein concentration c_{eq} in the separated supernatant was determined by UV-vis spectrophotometry using a NanoDrop™ (Thermo Fisher). The equilibrium fractional saturation q_{eq} was calculated as follows:

$$q_{\mathrm{eq}} = \left(c_0 - c_{\mathrm{eq}}\right) V_0\ /\ m_{\mathrm{HAP}}$$

The data (**Figure S5**) was fitted with a Langmuir isotherm from which the monolayer sorption saturation capacity X_{m} and the dissociation constant K_{D} were determined (a_{L}: Langmuir constant):

$$\mathrm{q} = (X_{\mathrm{m}} a_{\mathrm{L}}\, c_{\mathrm{eq}})\ /\ (1 + a_{\mathrm{L}}\, c_{\mathrm{eq}}) = \mathrm{X_m} c_{\mathrm{eq}}/(a_{\mathrm{L}}^{-1} + c_{\mathrm{eq}}) = \mathrm{X_m} c_{\mathrm{eq}}/(K_{\mathrm{D}} + c_{\mathrm{eq}})$$

The obtained dissociation constants K_{D} for OPN and OPNp adsorbing onto HAP nanoparticles are 28(17) µM and 14(4) µM, respectively; the monolayer sorption saturation capacities X_m are 0.011(3) µmol/mg and 0.006(1) µmol/mg, respectively.

S9 Table of chemical shift perturbations

The CSPs of all residues in OPN and OPNp upon interaction to Ca^{2+} (ratio 1:120 and saturated) and HAP can be found in a table on page S13.

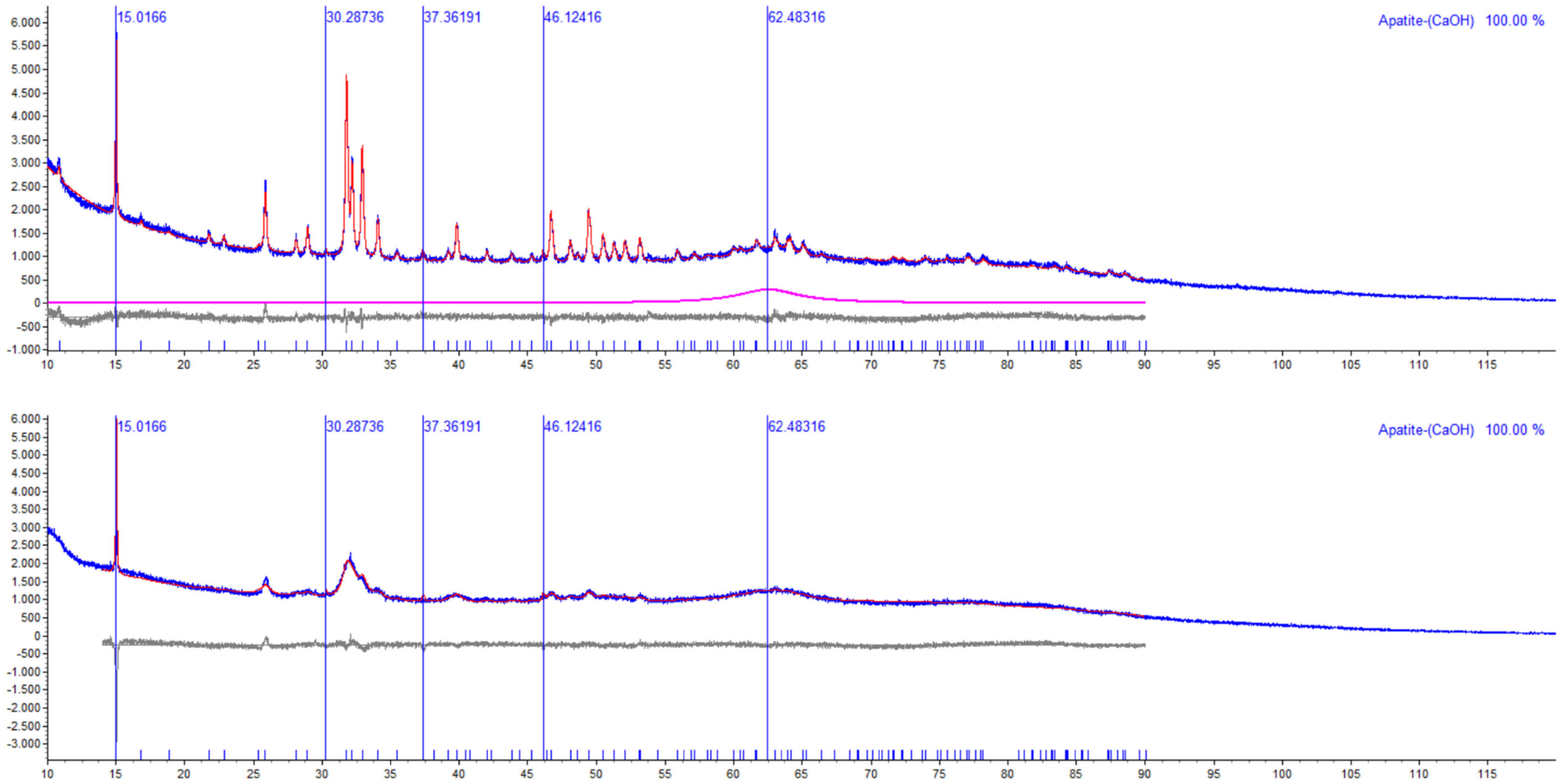

Figure S1. Rietveld refinement of the powder XRD patterns of the calcined HAP (top) and the HAP nanoparticles (bottom), including the observed intensities (blue), calculated intensities (red), amorphous peak (pink) and difference intensities (grey), using a hexagonal HAP with P63/m symmetry. Impurities and the corresponding reflection angles are marked with a blue bar.

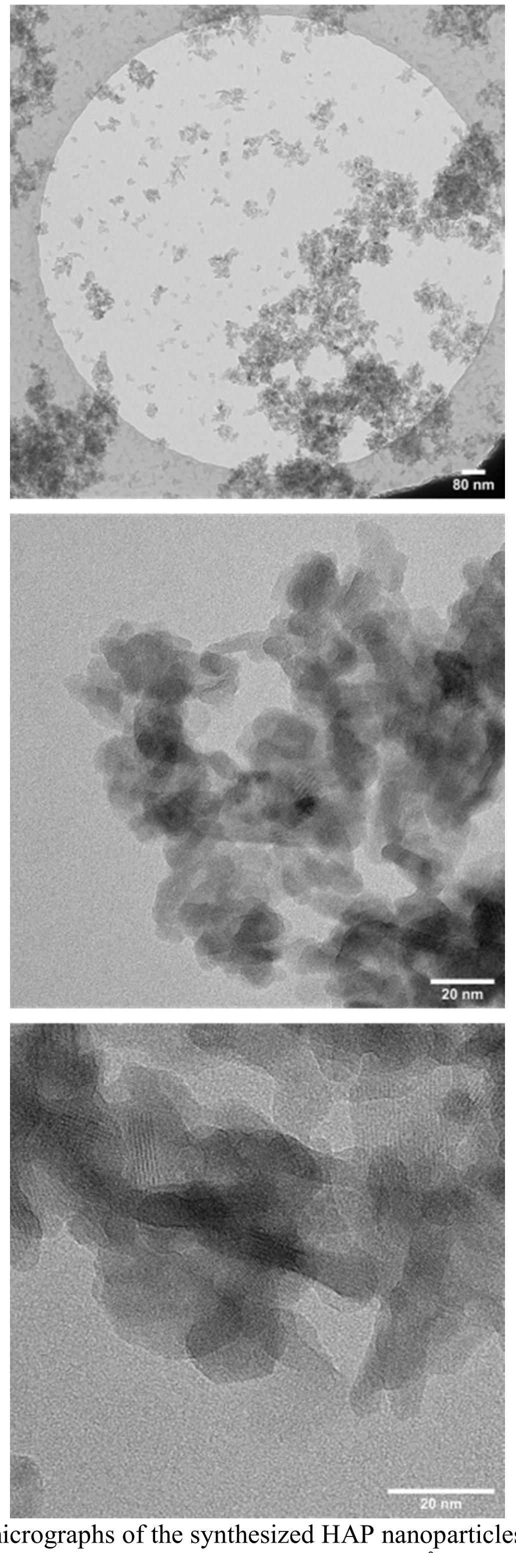

Figure S2. TEM micrographs of the synthesized HAP nanoparticles, recorded at three different magnifications with pixel size of 4.12, 0.38 and 0.27 Å, respectively (top to bottom).

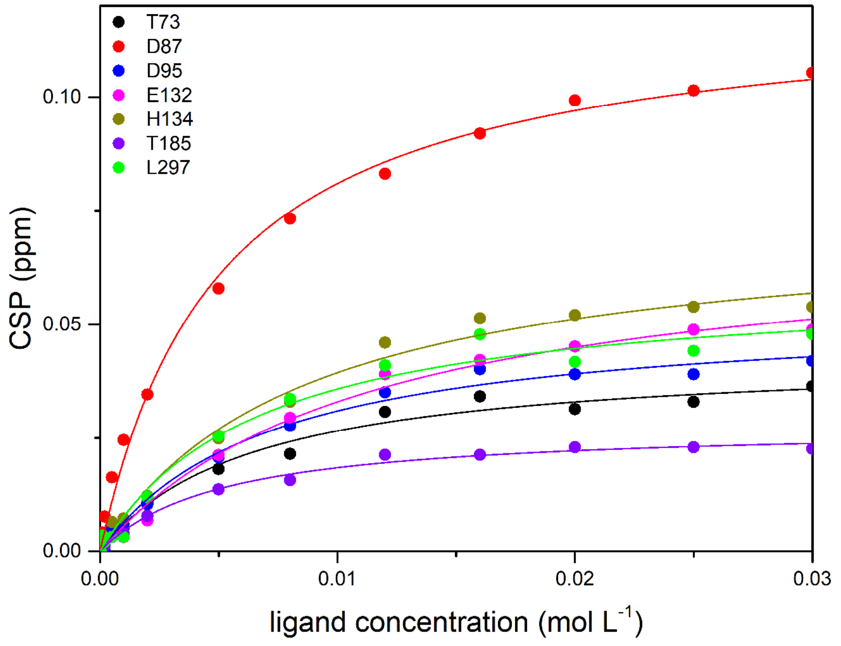

Figure S3. Selected titration curves from ^{1}H-^{15}N HSQC NMR spectra for ^{15}N-labelled OPN titrated with $CaCl_2$ for the residues T73, D87, D95, E132, H134, T185 and L297, together with the corresponding low-binding event fit (thin lines).

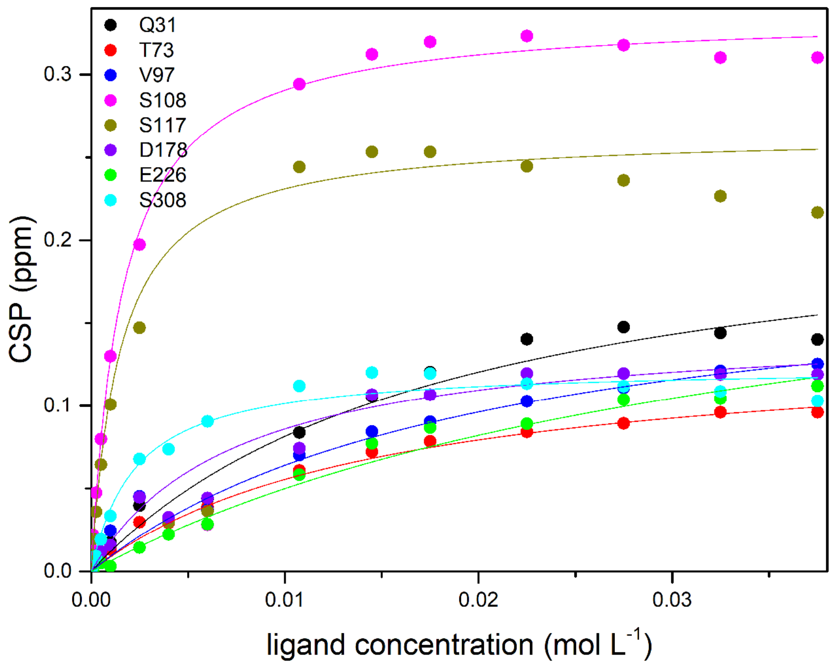

Figure S4. Selected titration curves from ^{1}H-^{15}N HSQC NMR spectra for ^{15}N-labelled OPNp titrated with $CaCl_2$ for the residues Q31, T73, V97, S108, S117, D178, E226 and S308, together with the corresponding low-binding event fit (thin lines).

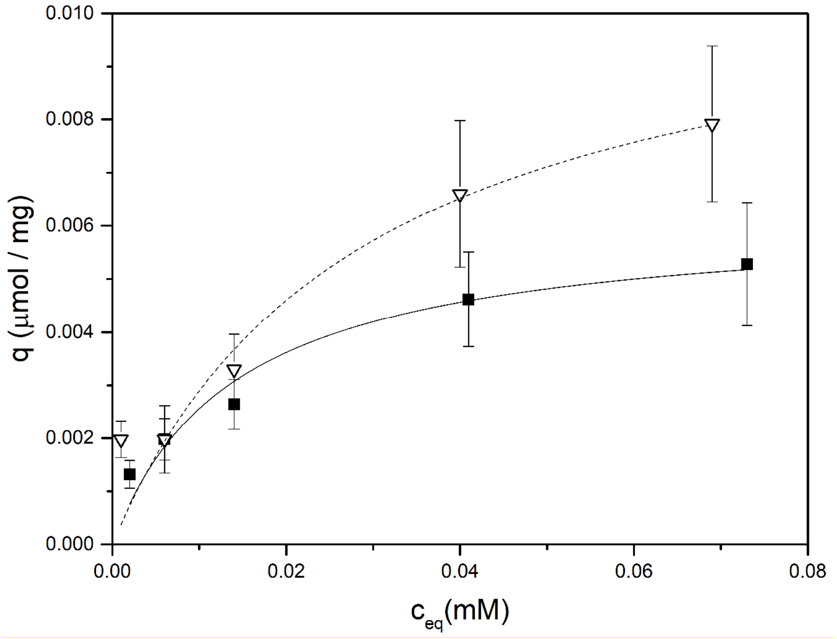

Figure S5. Adsorption isotherms for OPN (open triangles) and OPNp (filled squares) interacting with HAP, including standard deviations and corresponding fits, from which the monolayer sorption saturation capacities X_m and dissociation constants K_D were obtained (X_m (OPN) = 0.011(3) µmol/mg and K_D (OPN) = 28(17) µM; X_m (OPNp) = 0.006(1) µmol/mg and K_D (OPNp) = 14(4) µM).

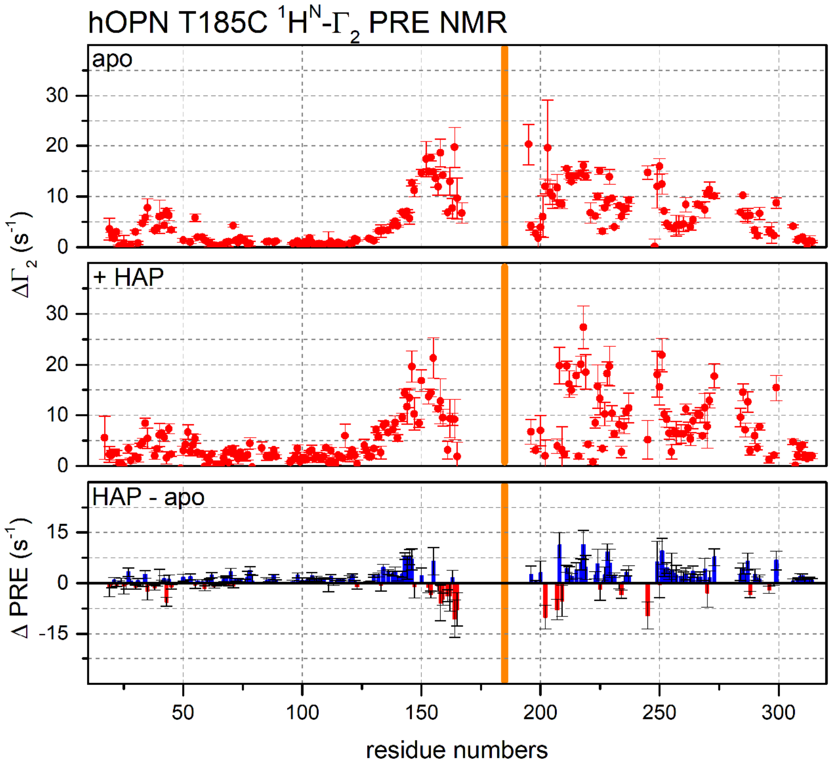

Figure S6. Effect of the interaction of the OPN cysteine mutant T185C with HAP on long-range interactions measured by $^1H^N$-T_2 NMR experiments. PRE profiles $\Delta\Gamma_2$ of the apo form (top) and OPN + HAP (middle). PRE rates difference plot Δ PRE = Γ_2(OPN_HAP)- Γ_2(OPN_apo) (bottom). Orange bars indicate the mutated cysteine residue with the attached MTSL label.

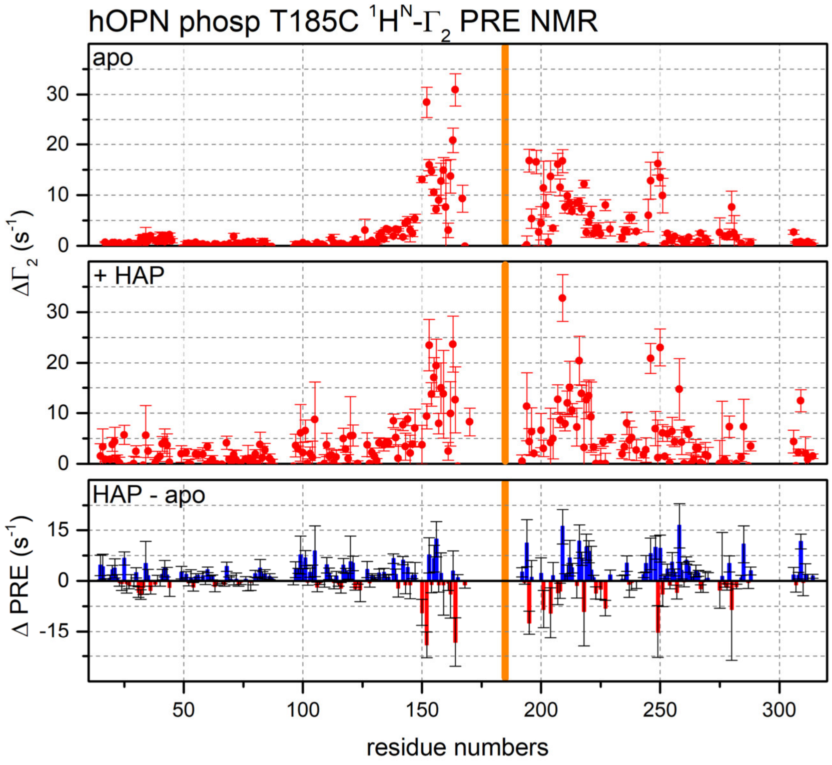

Figure S7. Effect of the interaction of the phosphorylated OPNp cysteine mutant T185C with HAP on long-range interactions measured by $^{1}H^{N}$-T_2 NMR experiments. PRE profiles $\Delta\Gamma_2$ of the apo form (top) and OPNp + HAP (middle). PRE rates difference plot Δ PRE = Γ_2(OPNp_HAP)- Γ_2(OPNp_apo) (bottom). Orange bars indicate the mutated cysteine residue with the attached MTSL label.

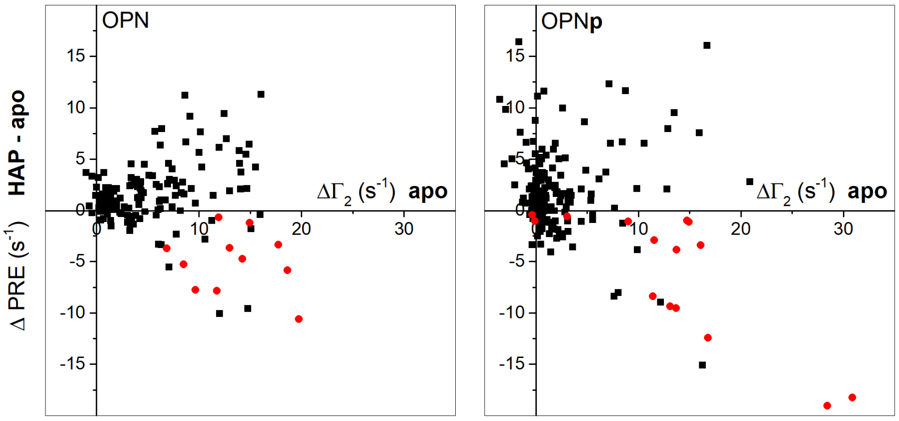

Figure S8. Correlation map of the PRE rates difference Δ PRE = Γ_2(OPN(p)_HAP)- Γ_2(OPN(p)_apo) and the PRE rates $\Delta\Gamma_2$ of the apo form of OPN (left) and OPNp (right). The red dots are from residues in the vicinity of the mutation site (residue 150-210).

References

[1] B. Mateos, J. Holzinger, C. Conrad-Billroth, G. Platzer, S. Żerkob, M. Sealey-Cardonac, D. Anrather, W. Koźmiński, R. Konrat, *Biochemistry* **2021**, *XX*, XXX-XXX**.**

[2] G. Platzer, S. Żerko, S. Saxena, W. Koźmiński, R. Konrat, *Biomol. NMR Assign.* **2015**, *9*, 289–292.

[3] V. S. Tagliabracci, J. L. Engel, J. Wen, S. E. Wiley, C. A. Worby, L. N. Kinch, J. Xiao, N. V. Grishin, J. E. Dixon, *Science* **2012**, *336*, 1150–1153.

[4] I. Matlahov, T. Iline-Vul, M. Abayev, E. M. Y. Lee, M. Nadav-Tsubery, K. Keinan-Adamsky, J. J. Gray, G. Goobes, *Chem. Mater.* **2015**, *27*, 5562–5569.

[5] A. X. S. Bruker, *TOPAS V4.2: General Profile and Structure Analysis Software for Powder Diffraction Data* **2009**, Karlsruhe, Germany.

[6] F. Delaglio, S. Grzesiek, G. W. Vuister, G. Zhu, J. Pfeifer, A. Bax, *J. Biomol. NMR* **1995**, *6*, 277–293.

[7] W. Lee, M. Tonelli und J. L. Markley, *Bioinformatics* **2014**, *31*, 1325-1327.

[8] M. P. Williamson, *Prog. Nucl. Magn. Reson. Spectrosc.* **2013**, *73*, 1-16.

S9 Table of chemical shift perturbations, including CSPs (in ppm) of all residues in OPN and OPNp upon interaction to Ca^{2+} (ratio 1:120 and saturated) and HAP.

OPN						OPNp				
residue	AA	CSP	CSP	CSP		residue	AA	CSP	CSP	CSP
		+ Ca2+ (1:120)	+ Ca2+ (1:300)	+ HAP				+ Ca2+ (1:120)	+ Ca2+ (1:750)	+ HAP
17	I	0.043	0.038	0.017		17	I	0.011	0.027	0.002
18	P					18	P			
19	V	0.000	0.001	0.000		19	V	0.031	0.030	0.003
20	K	0.003	0.003	0.000		20	K	0.004	0.012	0.001
21	Q	0.009	0.014	0.000		21	Q	0.021	0.062	0.006
22	A	0.004	0.001	0.004		22	A	0.017	0.042	0.002
23	D	0.005	0.007	0.005		23	D	0.034	0.096	0.005
24	S	0.008	0.014	0.013		24	pS	0.069	0.181	0.017
25	G	0.001	0.005	0.000		25	G	0.021	0.037	0.018
26	S	0.004	0.007	0.027		26	pS	0.021	0.011	0.048
27	S	0.002	0.001	0.009		27	pS	0.054	0.068	0.054
28	E	0.001	0.002	0.004		28	E	0.007	0.021	0.017
29	E					29	E	0.028	0.041	0.019
30	K	0.006	0.013	0.000		30	K	0.059	0.067	0.017
31	Q	0.010	0.025	0.004		31	Q	0.055	0.144	0.014
32	L	0.010	0.016	0.003		32	L	0.044	0.115	0.007
33	Y	0.011	0.016	0.005		33	Y	0.038	0.099	0.010
34	N	0.001	0.007	0.000		34	N	0.025	0.071	0.004
35	K	0.004	0.002	0.000		35	K	0.001	0.017	0.022
36	Y					36	Y	0.003	0.010	0.003
37	P					37	P			
38	D	0.009	0.008	0.003		38	D	0.002	0.012	0.003
39	A	0.003	0.009	0.000		39	A	0.005	0.009	0.007
40	V	0.003	0.001	0.000		40	V	0.007	0.003	0.000
41	A	0.000	0.002	0.000		41	A	0.006	0.006	0.004
42	T	0.003	0.003	0.004		42	T	0.003	0.003	0.004
43	W	0.003	0.003	0.005		43	W	0.005	0.009	0.004
44	L	0.005	0.006	0.004		44	L	0.009	0.018	0.002
45	N	0.009	0.004	0.004		45	N	0.002	0.017	0.000
46	P					46	P			
47	D					47	D			
48	P					48	P			
49	S	0.001	0.005	0.000		49	S	0.005	0.005	0.000
50	Q	0.012	0.021	0.006		50	Q			
51	K	0.003	0.006	0.000		51	K	0.012	0.009	0.004
52	Q	0.003	0.024	0.000		52	Q	0.006	0.009	0.000
53	N	0.006	0.009	0.006		53	N	0.010	0.011	0.004
54	L	0.006	0.006	0.011		54	L	0.006	0.015	0.005
55	L	0.012	0.012	0.004		55	L	0.017	0.031	0.006
56	A	0.004	0.006	0.004		56	A	0.010	0.018	0.002

57	P					57	P			
58	Q	0.005	0.007	0.003		58	Q	0.016	0.028	0.006
59	N	0.009	0.009	0.000		59	N	0.012	0.011	0.000
60	A	0.005	0.009	0.000		60	A	0.024	0.041	0.012
61	V	0.005	0.005	0.003		61	V	0.010	0.027	0.010
62	S	0.008	0.012	0.004		62	pS	0.025	0.069	0.047
63	S	0.002	0.005	0.002		63	pS	0.119	0.173	0.086
64	E	0.005	0.008	0.003		64	E	0.032	0.048	0.003
65	E					65	E			
66	T	0.013	0.019	0.000		66	T	0.035	0.080	0.002
67	N	0.004	0.003	0.007		67	N	0.014	0.021	0.006
68	D	0.011	0.012	0.000		68	D	0.022	0.031	0.002
69	F	0.007	0.009	0.001		69	F	0.014	0.024	0.006
70	K	0.029	0.037	0.009		70	K	0.028	0.015	0.008
71	Q	0.013	0.010	0.014		71	Q	0.011	0.019	0.000
72	E	0.018	0.015	0.003		72	E	0.034	0.056	0.006
73	T	0.031	0.036	0.010		73	T	0.050	0.092	0.019
74	L	0.017	0.023	0.006		74	L	0.039	0.077	0.014
75	P					75	P			
76	S	0.021	0.024	0.012		76	S	0.021	0.047	0.005
77	K	0.009	0.011	0.005		77	K	0.010	0.015	0.002
78	S	0.035	0.050	0.015		78	pS	0.057	0.165	0.025
79	N	0.013	0.015	0.014		79	N	0.013	0.077	0.004
80	E					80	E	0.008	0.039	0.025
81	S					81	pS	0.019	0.006	0.043
82	H					82	H	0.011	0.036	0.008
83	D	0.001	0.008	0.000		83	D	0.025	0.034	0.015
84	H	0.004	0.014	0.037		84	H	0.023	0.038	0.013
85	M	0.034	0.043	0.007		85	M			
86	D	0.022	0.036	0.022		86	D	0.009	0.012	0.008
87	D	0.083	0.105	0.054		87	D	0.019	0.028	0.021
88	M	0.049	0.064	0.030		88	M			
89	D	0.030	0.043	0.009		89	D			
90	D					90	D			
91	E					91	E			
92	D					92	D			
93	D					93	D			
94	D					94	D			
95	D	0.035	0.042	0.009		95	D			
96	H	0.026	0.033	0.015		96	H	0.073	0.126	0.036
97	V	0.030	0.039	0.022		97	V	0.082	0.135	0.005
98	D	0.012	0.008	0.005		98	D	0.037	0.050	0.011
99	S	0.020	0.023	0.015		99	pS	0.041	0.011	0.051
100	Q	0.020	0.023	0.005		100	Q	0.022	0.022	0.014
101	D	0.005	0.015	0.007		101	D	0.044	0.052	0.025
102	S	0.041	0.046	0.021		102	S	0.039	0.034	0.020
103	I	0.017	0.016	0.007		103	I	0.047	0.067	0.025

104	D	0.058	0.059	0.027		104	D	0.028	0.029	0.010
105	S	0.014	0.019	0.008		105	pS	0.095	0.025	0.053
106	N	0.023	0.030	0.008		106	N	0.023	0.029	0.032
107	D	0.000	0.016	0.006		107	D	0.051	0.093	0.013
108	S	0.039	0.058	0.017		108	pS	0.227	0.287	0.085
109	D	0.023	0.031	0.008		109	D	0.028	0.064	0.045
110	D	0.011	0.018	0.009		110	D	0.008	0.006	0.025
111	V	0.020	0.020	0.013		111	V	0.047	0.047	0.026
112	D	0.033	0.038	0.007		112	D	0.008	0.017	0.016
113	D	0.038	0.037	0.014		113	D	0.030	0.085	0.034
114	T	0.033	0.032	0.013		114	T	0.080	0.112	0.043
115	D	0.014	0.020	0.013		115	D	0.031	0.048	0.002
116	D	0.036	0.041	0.012		116	D	0.043	0.045	0.029
117	S	0.001	0.005	0.006		117	pS	0.165	0.186	0.092
118	H	0.003	0.009	0.008		118	H	0.036	0.065	0.023
119	Q					119	Q	0.029	0.052	0.013
120	S	0.008	0.008	0.004		120	pS	0.077	0.076	0.059
121	D	0.012	0.016	0.004		121	D	0.050	0.047	0.058
122	E	0.024	0.025	0.009		122	E	0.040	0.061	0.012
123	S	0.028	0.026	0.005		123	pS	0.009	0.031	0.036
124	H	0.025	0.043	0.017		124	H	0.007	0.019	0.017
125	H					125	H	0.023	0.074	0.016
126	S	0.009	0.002	0.012		126	pS	0.054	0.083	0.049
127	D	0.014	0.014	0.005		127	D	0.021	0.020	0.006
128	E	0.012	0.022	0.012		128	E	0.024	0.064	0.017
129	S	0.004	0.009	0.008		129	pS	0.064	0.010	0.044
130	D	0.008	0.012	0.011		130	D	0.010	0.036	0.018
131	E	0.005	0.010	0.000		131	E	0.059	0.039	0.019
132	L	0.039	0.049	0.006		132	L	0.078	0.144	0.020
133	V	0.013	0.011	0.012		133	V	0.019	0.036	0.007
134	T	0.046	0.054	0.014		134	T	0.058	0.102	0.016
135	D	0.009	0.010	0.003		135	D	0.011	0.022	0.000
136	F	0.009	0.011	0.002		136	F	0.030	0.043	0.013
137	P					137	P			
138	T	0.027	0.035	0.009		138	T	0.033	0.060	0.010
139	D	0.005	0.010	0.000		139	D	0.017	0.009	0.024
140	L	0.005	0.002	0.005		140	L	0.017	0.020	0.004
141	P					141	P			
142	A	0.010	0.008	0.000		142	A	0.063	0.112	0.004
143	T	0.014	0.021	0.006		143	T	0.035	0.075	0.004
144	E	0.004	0.005	0.004		144	E	0.021	0.034	0.000
145	V	0.007	0.007	0.003		145	V	0.027	0.056	0.004
146	F	0.008	0.013	0.005		146	F	0.019	0.026	0.008
147	T	0.008	0.007	0.006		147	T	0.020	0.041	0.000
148	P					148	P			
149	V	0.003	0.009	0.004		149	V			
150	V	0.010	0.009	0.009		150	V	0.031	0.054	0.005

151	P					151	P			
152	T	0.007	0.005	0.003		152	T	0.020	0.032	0.009
153	V	0.004	0.017	0.004		153	V	0.021	0.033	0.000
154	D	0.004	0.015	0.000		154	D	0.021	0.032	0.008
155	T	0.002	0.013	0.000		155	T	0.006	0.009	0.003
156	Y	0.002	0.006	0.000		156	Y	0.006	0.009	0.000
157	D	0.004	0.009	0.000		157	D	0.035	0.022	0.005
158	G	0.002	0.006	0.003		158	G	0.004	0.007	0.003
159	R	0.008	0.014	0.005		159	R	0.006	0.018	0.005
160	G	0.013	0.025	0.003		160	G	0.003	0.030	0.000
161	D	0.011	0.009	0.003		161	D	0.001	0.017	0.004
162	S	0.005	0.002	0.000		162	S	0.003	0.005	0.005
163	V	0.009	0.007	0.008		163	V	0.009	0.011	0.002
164	V	0.009	0.002	0.011		164	V	0.034	0.036	0.015
165	Y	0.011	0.006	0.013		165	Y	0.033	0.054	0.010
166	G	0.003	0.010	0.008		166	G	0.020	0.023	0.010
167	L	0.003	0.010	0.000		167	L	0.005	0.008	0.000
168	R					168	R	0.010	0.026	0.017
169	S					169	S	0.042	0.092	0.019
170	K					170	K	0.015	0.044	0.010
171	S					171	S	0.038	0.070	0.012
172	K					172	K	0.019	0.051	0.012
173	K					173	K	0.021	0.020	0.009
174	F					174	F	0.081	0.066	0.004
175	R					175	R			
176	R					176	R			
177	P					177	P			
178	D	0.012	0.045	0.026		178	D	0.083	0.188	0.017
179	I	0.005	0.023	0.021		179	I	0.044	0.138	0.017
180	Q	0.003	0.010	0.010		180	Q	0.017	0.055	0.002
181	Y	0.008	0.004	0.016		181	Y	0.016	0.017	0.005
182	P					182	P			
183	D	0.002	0.005	0.006		183	D	0.022	0.060	0.002
184	A	0.006	0.032	0.009		184	A	0.029	0.117	0.012
185	T	0.021	0.023	0.007		185	pT	0.034	0.253	0.098
186	D	0.011	0.020	0.004		186	D			
187	E	0.054	0.048	0.046		187	E			
188	D	0.006	0.011	0.003		188	D	0.016	0.043	0.004
189	I	0.007	0.009	0.005		189	I	0.021	0.012	0.006
190	T	0.004	0.010	0.010		190	T	0.008	0.024	0.010
191	S	0.003	0.011	0.012		191	pS	0.071	0.040	0.052
192	H	0.024	0.040	0.007		192	H	0.009	0.010	0.004
193	M					193	M	0.057	0.052	0.019
194	E	0.008	0.001	0.018		194	E	0.003	0.021	0.009
195	S	0.003	0.010	0.008		195	pS	0.017	0.017	0.059
196	E	0.011	0.015	0.004		196	E	0.033	0.069	0.011
197	E					197	E	0.021	0.038	0.022

198	L	0.004	0.013	0.006		198	L	0.016	0.035	0.006
199	N	0.008	0.019	0.011		199	N	0.010	0.032	0.006
200	G	0.003	0.011	0.005		200	G	0.004	0.008	0.007
201	A	0.003	0.011	0.000		201	A	0.005	0.015	0.000
202	Y	0.005	0.013	0.000		202	Y	0.004	0.023	0.005
203	K	0.010	0.009	0.008		203	K	0.011	0.018	0.003
204	A	0.004	0.004	0.004		204	A	0.004	0.009	0.002
205	I	0.008	0.010	0.004		205	I	0.020	0.030	0.009
206	P					206	P			
207	V	0.006	0.006	0.000		207	V	0.020	0.026	0.005
208	A	0.012	0.017	0.002		208	A	0.034	0.054	0.012
209	Q	0.014	0.012	0.004		209	Q	0.020	0.037	0.007
210	D	0.004	0.014	0.000		210	D	0.016	0.019	0.003
211	L	0.005	0.010	0.006		211	L	0.007	0.012	0.004
212	N	0.001	0.010	0.000		212	N	0.033	0.036	0.010
213	A	0.003	0.002	0.002		213	A	0.036	0.046	0.008
214	P					214	P			
215	S	0.013	0.020	0.000		215	pS	0.022	0.039	0.011
216	D	0.006	0.012	0.006		216	D	0.012	0.019	0.005
217	W	0.006	0.010	0.007		217	W	0.013	0.048	0.010
218	D	0.005	0.004	0.000		218	D	0.027	0.052	0.010
219	S	0.007	0.006	0.002		219	pS	0.049	0.044	0.082
220	R	0.008	0.009	0.001		220	R	0.017	0.006	0.009
221	G	0.000	0.005	0.006		221	G	0.014	0.027	0.006
222	K	0.012	0.008	0.010		222	K	0.002	0.028	0.000
223	D	0.006	0.004	0.000		223	D	0.014	0.014	0.010
224	S	0.006	0.013	0.001		224	pS	0.048	0.039	0.068
225	Y	0.003	0.017	0.003		225	Y	0.018	0.049	0.014
226	E	0.003	0.015	0.004		226	E	0.049	0.097	0.008
227	T	0.005	0.006	0.000		227	T	0.029	0.032	0.010
228	S	0.007	0.007	0.010		228	pS	0.011	0.072	0.041
229	Q	0.006	0.008	0.004		229	Q	0.025	0.042	0.008
230	L	0.010	0.015	0.005		230	L			
231	D	0.005	0.013	0.004		231	D			
232	D					232	D			
233	Q	0.004	0.006	0.007		233	Q			
234	S	0.007	0.003	0.006		234	pS	0.076	0.066	0.052
235	A	0.009	0.014	0.004		235	A	0.016	0.039	0.013
236	E	0.005	0.011	0.006		236	E	0.023	0.052	0.019
237	T	0.003	0.005	0.007		237	T	0.018	0.047	0.020
238	H					238	H	0.021	0.033	0.004
239	S					239	S	0.014	0.024	0.004
240	H					240	H	0.000	0.029	0.006
241	K					241	K			
242	Q					242	Q			
243	S					243	S	0.024	0.028	0.007
244	R					244	R	0.015	0.020	0.012

245	L	0.028	0.036	0.000		245	L	0.006	0.026	0.008
246	Y	0.017	0.025	0.007		246	Y	0.013	0.046	0.010
247	K	0.021	0.035	0.006		247	K	0.003	0.028	0.007
248	R	0.004	0.004	0.008		248	R	0.010	0.015	0.003
249	K	0.003	0.006	0.008		249	K	0.008	0.022	0.000
250	A	0.002	0.007	0.004		250	A	0.014	0.027	0.007
251	N	0.011	0.022	0.012		251	N	0.009	0.037	0.001
252	D	0.014	0.020	0.002		252	D	0.012	0.024	0.006
253	E	0.011	0.024	0.004		253	E	0.013	0.058	0.010
254	S	0.004	0.007	0.008		254	pS	0.041	0.031	0.080
255	N	0.009	0.030	0.017		255	N	0.030	0.057	0.020
256	E	0.004	0.005	0.005		256	E	0.008	0.008	0.007
257	H	0.027	0.037	0.012		257	H	0.014	0.025	0.005
258	S	0.014	0.023	0.015		258	pS	0.016	0.023	0.008
259	D	0.002	0.002	0.007		259	D	0.007	0.011	0.002
260	V	0.004	0.002	0.005		260	V	0.070	0.087	0.016
261	I	0.006	0.003	0.007		261	I	0.071	0.127	0.022
262	D	0.005	0.006	0.004		262	D	0.006	0.040	0.007
263	S	0.006	0.014	0.004		263	pS	0.025	0.050	0.064
264	Q	0.002	0.006	0.010		264	Q	0.011	0.009	0.006
265	E					265	E	0.010	0.016	0.009
266	L	0.004	0.012	0.004		266	L	0.021	0.016	0.006
267	S	0.017	0.031	0.005		267	S	0.013	0.019	0.000
268	K	0.006	0.009	0.003		268	K	0.029	0.043	0.001
269	V	0.007	0.014	0.000		269	V	0.035	0.062	0.005
270	S	0.007	0.006	0.014		270	pS	0.035	0.107	0.029
271	R	0.004	0.005	0.017		271	R			
272	E					272	E			
273	F	0.024	0.027	0.013		273	F			
274	H					274	H			
275	S					275	pS	0.031	0.071	0.024
276	H					276	H	0.027	0.047	0.010
277	E					277	E	0.014	0.018	0.015
278	F					278	F	0.017	0.022	0.020
279	H					279	H	0.036	0.018	0.000
280	S					280	pS	0.031	0.115	0.020
281	H					281	H	0.041	0.105	0.016
282	E					282	E	0.044	0.043	0.011
283	D					283	D			
284	M	0.007	0.010	0.007		284	M	0.018	0.035	0.001
285	L	0.012	0.020	0.000		285	L	0.047	0.097	0.010
286	V	0.011	0.014	0.004		286	V	0.009	0.006	0.006
287	V	0.001	0.001	0.004		287	V	0.030	0.045	0.008
288	D	0.002	0.002	0.001		288	D	0.020	0.031	0.004
289	P					289	P			
290	K	0.005	0.009	0.004		290	K			
291	S	0.004	0.006	0.003		291	S			

292	K	0.007	0.009	0.003		292	K			
293	E					293	E			
294	E					294	E			
295	D					295	D			
296	K	0.014	0.013	0.014		296	K			
297	H	0.041	0.048	0.010		297	H			
298	L	0.003	0.006	0.000		298	L			
299	K	0.033	0.064	0.008		299	K			
300	F					300	F			
301	R					301	R			
302	I					302	I			
303	S					303	S			
304	H					304	H			
305	E					305	E			
306	L	0.014	0.020	0.006		306	L	0.049	0.066	0.015
307	D	0.035	0.039	0.016		307	D	0.027	0.023	0.022
308	S	0.004	0.016	0.002		308	pS	0.060	0.047	0.114
309	A	0.005	0.014	0.007		309	A	0.090	0.159	0.025
310	S	0.008	0.009	0.004		310	pS	0.089	0.065	0.103
311	S	0.009	0.011	0.006		311	pS	0.024	0.041	0.016
312	E	0.005	0.005	0.002		312	E	0.025	0.048	0.000
313	V	0.009	0.011	0.004		313	V	0.044	0.084	0.020
314	N	0.012	0.023	0.002		314	N	0.021	0.049	0.007
